# The Acute Care for Elders Unit Model of Care

**DOI:** 10.3390/geriatrics3030059

**Published:** 2018-09-11

**Authors:** Robert M. Palmer

**Affiliations:** Internal Medicine, Eastern Virginia Medical School 825 Fairfax Avenue, Suite 201 Norfolk, VA 23507, USA; palmerrm@evms.edu

**Keywords:** acute hospital care, older adults, geriatric assessment, interdisciplinary team

## Abstract

Older patients are at risk for loss of self-care abilities during the course of an acute medical illness that results in hospitalization. The Acute Care for Elders (ACE) Unit is a continuous quality improvement model of care designed to prevent the patient’s loss of independence from admission to discharge in the performance of activities of daily living (hospital-associated disability). The ACE unit intervention includes principles of a prepared environment that encourages safe patient self-care, a set of clinical guidelines for bedside care by nurses and other health professionals to prevent patient disability and restore self-care lost by the acute illness, and planning for transitions of care and medical care. By applying a structured process, an interdisciplinary team completes a geriatric assessment, follows clinical guidelines, and initiates plans for care transitions in concert with the patient and family. Three randomized clinical trials and systematic reviews of ACE or related interventions demonstrate reduced functional disability among patients, reduced risk of nursing home admission, and lower costs of hospitalization. ACE principles could improve elderly care in any acute setting. The aim of this commentary is to describe the ACE model and the basis of its effectiveness.

## 1. Introduction

The Acute Care for Elders (ACE) model of care was designed to reduce the incidence of functional disability of older adults occurring during hospitalization for acute medical illness [1]. The ACE intervention was conducted on a medical–surgical unit (ACE Unit) with the objective of preventing loss of independence in the activities of daily living (ADL) and to restore by the time of discharge ADL independence lost during the course of the acute illness and hospitalization. The ACE intervention used complementary principles of continuous quality improvement and comprehensive geriatric assessment in developing a new system of care for acutely ill older adults [2]. A multi-dimensional intervention included four key elements: a physical environment designed to promote patient functional independence and safety, patient–centered care delivered at the bedside by registered nurses in collaboration with interdisciplinary providers, comprehensive discharge planning undertaken early in the hospitalization and informed by the interdisciplinary team, and medical care review to assure quality of medication prescribing and clinical management.

Previous publications have highlighted the risks of iatrogenic illness, adverse consequences of immobility and prolonged bedrest, and “the hazards of hospitalization” [3,4]. Studies of the natural history of functional morbidity in hospitalized older patients revealed the loss of independence from baseline physical functioning to hospital discharge in the majority of older adults and highlighted the adverse consequences of hospitalization [5,6]. Small clinical trials showed that modifications of the physical environment of the hospital and the deployment of interdisciplinary teams could significantly improve important clinical outcomes [1,7]. In creating the ACE model, investigators placed emphasis on making the environmental setting capable of safely supporting the patient’s personal needs and abilities. A patient-centered approach is facilitated by an interdisciplinary team notable for its skills at assessing and enhancing the physical functioning of acutely ill older adults. A funding opportunity from the John A. Hartford Foundation enabled the investigators to design, implement, and evaluate the ACE unit and measure its effectiveness on the outcomes of physical functioning measured as basic ADL [8], and to assess the impact of the intervention on secondary outcomes including costs of care, length of hospital stay, self–reported mobility, and the subsequent transition to nursing facilities or home. Although the cost of the intervention was not a primary outcome measure, the intervention was created to be ideally cost neutral. Three randomized clinical trials of the ACE unit were conducted with somewhat different objectives but with fidelity to key elements and pragmatic adaptations reflecting a continuous quality improvement intervention. The clinical trials compared usual care on medical–surgical units versus care on the ACE unit. 

The aim of this commentary is to describe the ACE model and the basis of its effectiveness. The conceptual basis for the ACE intervention, the clinical trials supporting the effectiveness of ACE versus usual care, the lessons learned from these experiences including barriers to wider dissemination of the ACE model, and future directions of acute care models are reviewed. These lessons are even more relevant today than they were in the 1990s when the ACE model was launched. The American population is aging, older adults have a higher rate of hospitalization and longer lengths of stay than middle-aged patients, chronic multiple conditions are present in most hospitalized older adults, procedural interventions in older adults are growing in numbers, and Medicare costs for hospitalization have steadily risen [9,10]. 

### Conceptual Basis

Acute illness is often a stressful experience for older adults and their family caregivers. Already acutely ill and usually with multiple chronic conditions, elements of hospitalization, such as a hostile (unsafe and anxiety producing) physical environment and processes of patient care (e.g., restricting mobility), may contribute to the patient’s loss of functional independence or a failure to restore physical functioning to baseline prior to the acute illness and hospitalization. In the original conceptual model of dysfunctional syndrome, now referred to as hospital-associated disability [11], impaired homeostatic reserves and multiple chronic conditions predispose the older patient to functional decline. Age-related loss of muscle mass, strength, and aerobic capacity may be further compromised by the deconditioning effects of immobility, postural instability, impaired baroreceptor reflexes (e.g., causing orthostatic hypotension or unsteadiness) and emerging physical impairments that might affect cognition leading to delirium, anxiety, or depressive symptoms. A further deleterious effect on patient functional status is the usual silo-based care that often excludes the patient or family caregivers from engagement in the patient’s recovery from illness and return to independent functioning [12]. Hospital-associated conditions, such as falls with injury, incident delirium, ischemic pressure ulcers, and catheter-associated urinary tract infections, are common in patients over age 65 but are potentially preventable. The hostile environment, prolonged bedrest, undernutrition/starvation, immobility/physical restraints/tethers, and depersonalization contribute to the risks of functional decline and culminate in a dysfunctional or disabled patient. However, any disruption to this functional decline could potentially improve hospital outcomes by mitigating the adverse effects of the hospital experience, such as by enabling patients to walk, initiate self-care, socialize with other patients and family members, obtain adequate sleep, and achieve adequate nutritional intake [13]. In particular, immobility has potentially devastating effects on the patient’s functional status, and freedom to perform ADL such as transferring from bed to chair and walking. The ACE Unit was created to systemically disrupt or reverse hospital-associated disability while making patient throughput more efficient and patient-centered.

## 2. Development of the ACE Unit

The ACE unit intervention represents a major cultural change in the hospital care of older adults. Focus of care is on the patient and not on single diseases, and the full intervention requires adaptation of the physical environment to enhance patient functional independence and safety, and a shift from multidisciplinary to interdisciplinary team-based care. This transformation is facilitated by a business strategy that appeals to the hospital’s corporate leadership as important stakeholders and demonstrates the value to the institution (e.g., return on investment) and its marketable reputation. A step-by-step approach informs components of an ACE unit and advises a potential business strategy [14,15]. However, without buy-in from the corporate suite the costs of transforming the nursing unit and retraining of staff can prove prohibitive. Although research has shown that ACE units do not increase costs of hospitalization [16], there can be initial costs related to retraining of professional staff and upgrading/remodeling of older nursing units. The first ACE unit, at University Hospitals of Cleveland, for example, was an older medical–surgical unit with 15 beds allotted to geriatric patients. The hospital unit was in need of modernization and, with extramural funding, substantial changes in the physical structure of the unit were made in order to create the prepared environment. (However, much of the structural redesign of the first ACE Unit is standard design in new or upgraded community hospitals today.)

Once leadership agrees to form an ACE unit, an advisory council consisting of leaders from each department relevant to geriatric care is formed to guide the development of the program and to set the stage for interdisciplinary team care. In the original ACE unit, each director had direct input into the creation of patient guidelines for optimal care of complex older adults. An advisory council also helps to create close collaboration among team members and advocates for the quality care of older patients [15,17]. Thereafter, the advisory council meets periodically to review progress on the ACE unit and to review metrics that justify the return on investment, such as hospital costs, quality metrics, and prevention of hospital-acquired conditions. By building this collaboration, all health care providers share in ownership of the ACE units and, as advocates, help to sustain and grow the ACE program. 

### 2.1. The Prepared Environment

Design of the first ACE unit was informed by experiences in architectural design of acute-care and long-term care facilities, inpatient rehabilitation programs, and prior experience of clinicians and researchers [1]. To make the physical environment more home-like and minimize the risks of falls, confusion, anxiety, and deconditioning, the original unit was carpeted, handrails were installed in hallway corridors, the hallways were uncluttered, and wallpaper and paint colors emphasized earth tones with contrasts between floor, wall, and ceiling to aid patients with impaired depth perception [17]. Diffuse lighting including wall sconces and wall lighting behind the patient’s bed was added, door handle levers were installed, large clocks and calendars were added to help with orientation, bathrooms had elevated toilet seats, and wall hangings and carpeting helped to reduce ambient noise [1,2]. A designated space was created to encourage socialization of patients with other patients and family members. While these transformations were innovations in the 1990s, many hospitals today include these structural elements, except for carpeting, preferring compressible synthetic flooring. A greater focus on wellness, calming environments, privacy, and noise reduction is standard procedure in new hospitals, reflecting marketing forces, and hopes to enhance patient satisfaction and improve patient safety [18]. As hospital designs become more “senior friendly,” the nursing units become more closely aligned with ACE principles of the prepared environment, thereby obviating the need to transform medical-surgical units as was a necessary cost in the earlier ACE Units. Hospitals today must comply with the Americans with Disabilities Act (https://www.ada.gov/) with room specifications that are quite compatible with the prepared environment of earlier ACE Units (Table 1). Furthermore, the redesign of nursing units is likely to be acceptable to most adult patients implying that future designs of medical units for adults should resemble the ACE Unit. The prepared environment is also an example of a safety culture where the priority to prevent hospital-acquired conditions merges with the prevention of immobility, acute confusion, and geriatric syndromes. The argument can be made that the design of ACE Units should be standard practice when constructing new hospitals and upgrading nursing units [19]. 

### 2.2. Patient-Centered Care

Patient-centered care is defined as “providing care that is respectful of, and responsive to, individual patient preferences, needs and values, and ensuring that patient values guide all clinical decisions” [20]. Patient-centered care considers patients’ cultural traditions, their personal preferences and values, and the needs of their family members, and includes them as integral members of the interdisciplinary team when making clinical decisions. In ACE units, healthcare providers with a vested interest in the care of older adults share responsibility with the attending clinician/physician for management of the patient and interactions with other team members. In the model of interdisciplinary team-based care, health professionals coordinate care and communicate with one another as well as directly with the patient and family. Unlike traditional silo-based and multidisciplinary care, the team members in the ACE unit prioritize what professional services are needed and create greater efficiencies than are normally seen in usual care. Recommendations are based on agreed-upon standards of practice for patients on the ACE unit. 

The ACE unit model benefits from the pivotal role of nurses who provide 24/7 bedside care, advanced practice nurses who typically oversee interdisciplinary rounds and connect with attending physicians and consultant geriatricians daily. Most ACE units have trained geriatric resource nurses and many are affiliated with Nurses Improving Care of Healthcare Systems for Elders (NICHE) [21]. Under the supervision of the advance practice nurse and, with consultation provided by geriatric resource nurses, primary bedside nurses are instructed in guidelines (protocols) for the bedside care of older adults. These protocols include preventative measures designed to prevent decline in the patient’s performance of ADL: bathing, dressing, transferring from bed to chair, toileting, and feeding. For patients who are already impaired in the ADL, restorative guidelines are designed to help patients regain independent functioning and to inform further evaluation by physical and occupational therapists or care managers. The protocols weight heavily on patient mobility, ADL functioning, patient nutrition with specific goals, and attention to skin integrity, urinary and bowel continence, cognitive function (including maintaining/restoring normal wake and sleep cycles, and prevention of delirium), and augmenting hearing and vision [17]. 

The major contributions of team members during interdisciplinary rounds are shown in Table 2. Typically, daily meetings last 30 min with a conservative goal of reviewing 10 patients including all new admissions. On average, 5 min are spent in discussion of new patients and 1–2 min for follow-up patients. Realistically not all members of the healthcare team can attend daily rounds. The core team includes the bedside nurse, the clinical nurse specialist/geriatric resource nurse, and the care manager. Other team members as available include the attending clinician, geriatrician if present, and clinical pharmacist. Extended team members as available include the physical and occupational therapists, dietitian, and speech-language therapist. The advance practice nurse serves as the “quarterback” in touching base with all members of the team in order to assure consistency of the care plans and to update patients and providers. Team rounds help to reinforce bedside nursing care and to clarify uncertain goals such as patient preferences, prognosis, and specialty consult recommendations. Geriatricians, on occasion, mediate differences in care plans between the attending clinician and the interdisciplinary team or specialty physicians. The ACE principles of patient-centeredness, assuring patient safety, and independent physical functioning are foundational to the recommendations made by the team members. 

A functional trajectory is created by the interdisciplinary team during rounds that identifies the patient’s current functional status, baseline functional status typically anchored as 2 weeks prior to admission, and social support network prior to the acute illness leading to hospitalization [22]. The functional trajectory includes review of baseline function at the time of hospital admission in the patient’s independent performance of baseline ADL and instrumental ADL, mobility, cognitive function, mood and affect, living situation, and social supports, and presence of an advanced directive. 

The baseline functional status is contrasted to the admission functional assessment based on the nurse and attending clinician’s evaluation of basic ADL, cognition (delirium, dementia), current mood and affect such as anxiety or depression, and nutritional status based on oral food and fluid consumption.

The interdisciplinary team implements patient-centric care that may include physical therapy, occupational therapy, medication review by a geriatrician or pharmacist, nutritional support, and care coordination. The team establishes a goal of having the patient return to their baseline physical functioning by the time of hospital discharge.

The daily goal is to enhance the patient’s independent performance of ADL and mobility, and to assure the patient is clinically stable and clinically capable of safe return to home prior to the time of discharge, or has sufficient social supports for safe caregiving. The alternative is to recommend a site such as a postacute facility for rehabilitation or a long-term care facility. The functional trajectory was reviewed daily in team rounds and plans are modified as necessary.

### 2.3. Pivotal Importance of Physical Functioning

The relationship of baseline functioning to the subsequent transition to postacute care was validated in a secondary analysis of the first ACE unit intervention trial [23]. The team realized that the oldest patients, notably those aged 90 years and older, were most likely to experience a decline in performance of basic ADL, as documented in a subsequent analysis [24].

Particular attention was paid to cognition with a realization of the risk of functional decline related to prevalent delirium [25].

Recognition of depression, although challenging with acutely ill and complex older patients, is considered important to the team and was shown to be associated with functional decline and increased risk of 3-year mortality [26,27].

Severe medical illness and multiple chronic conditions often inform the interdisciplinary team of the patient’s prognosis and appropriateness of transitioning from aggressive interventions to comfort care only or hospice.

The team-initiated family/patient conferences are held when faced with uncertain clinical pathways, a transition to comfort measures, or review of the patient’s goals of care. Subsequent analysis of ACE cohorts identified six independent predictors of 1-year mortality among survivors of hospitalization that included ADL function and the predictive index has become a widely disseminated tool [28]. In all ACE units, the geriatric assessment of ADL function, mood and cognition, and acute and comorbid medical conditions, was the initial responsibility of the attending clinician and bedside nurse. The bedside nurse today benefits from training as a geriatric resource nurse and participation in the NICHE online nodules [21].

Arguably, the most important bedside assessment made by the nurse and team is mobility status as mobility determines the patient’s ability to perform ADL, bear weight and walk, or use assistive devices. Low mobility during hospitalization has been correlated with functional decline in older hospitalized adults [29] and the functional impact of bed rest even in healthy older adults has been demonstrated with a rapid decline in muscle strength and mass [30]. Immobility has long been recognized as a hazard of hospitalization [4]. Acutely ill older adults are often exhausted or cognitively impaired and unable to participate in physical activities. Nurses, using guidelines, will perform a passive or active range of motion exercises with the patient and turn the patient frequently in order to maintain adequate skin integrity and conditioning until the patient can be mobilized further such as with a lift or strap to assist them during transfers from bed to chair. In some hospitals and ACE units, a lift team is ordered in order to facilitate patient transfers. Although extraordinary efforts are required at times to improve patient mobility, the benefits to nursing staff are downstream as the patient will be less likely to develop complications of immobility and persistent bedrest. For example, risk factors for urinary catheterization in medically ill patients are well described, including immobility, as are the adverse consequences of persistent catheterization, including urinary tract colonization or infection, in the absence of a specific indication [31,32]. Patients who report unsteadiness at hospital admission are also more likely to experience functional decline by the time of hospital discharge [33]. Subsequent studies of patients enrolled in ACE unit clinical trials has shed light on the importance of independent physical functioning during and subsequent to hospitalization. In one analysis, the failure to recover independence in baseline ADL that was lost since hospitalization was associated with an increased risk of further decline in ADL functioning, institutionalization, and death [34]. Using data from ACE unit trials, investigators have created a clinical index to stratify hospitalized older patients according to their risk for new onset ADL disability. There are 10 items that are available to the ACE team within 24 h of the patient’s admission that are independently associated with a loss of independence in one or more ADL and that in the aggregate highly predict functional decline by hospital discharge [35]. By combining databases from more than one ACE unit study, prediction of recovery of ADL independence, dependence or death in elders who become disabled during hospitalization can be estimated [36]. This information provides important clinical value to the physician and clinical team caring for the patient, particularly when approaching difficult conversations with patients regarding the goals of care and prognosis. Although these indices have not been studied prospectively in clinical trials, the findings are robust and imply their value as clinical tools.

### 2.4. Planning for Transition to Home (Discharge Planning)

In ACE units, the process of planning for the patient’s transition back home begins on the first day of admission. The assumption made by the interdisciplinary team is that if the patient was living at home, then the goal is to have them return home guided by the functional trajectory. The original ACE unit studies were not focused on postacute transitions of care, but the team understood that patient clinical stability and physical functioning were keys to a safe transition. The patient-centric components of the ACE intervention are especially important in reducing the risks of postacute placement or early unplanned hospital readmission. Limitations in funding from extramural sources and limited postacute resources prevented the ACE unit studies from including interventions focused on postacute transitions of care from the unit. However, other investigators whose research focused on transitions of care created evidence-based models, that in conjunction with the ACE unit intervention, could have potentially greater benefits for patients and produce reduced rates of hospital readmissions [37,38]. Importantly, acute care and transitional care interventions have been adopted by hospitalists in quality improvement programs.

### 2.5. Medical Care Review

In many ACE units, the major role of a geriatrician or an advanced practice nurse is to educate and mentor interdisciplinary team members, the bedside nurses, support staff, and the attending physicians using techniques of academic detailing [39]. Medical review includes oversight of quality measures and metrics and assuring adherence to contemporary practice. Medical care review also helps mediate conflicts in the direction of care that might arise among team members or the team and specialty providers. For example, through engagement with the patient and family, the interdisciplinary team may have recommended comfort care and transition to hospice for a patient with an advanced serious illness and frailty, but subspecialists may recommend invasive procedures in a desperate effort to cure the patient of a single disease. The leader of the team might interface with the subspecialty provider and advocate for the patient and family whose views differ from the specialist’s. This advocacy role of the team leaders also applies when the patients are not receiving evidence-based treatments. For example, an attending physician might decline to prescribe oral anticoagulants to a patient with atrial fibrillation and risk factors for atheroembolic stroke because of a perceived greater risk of bleeding and injury from falls. Physician detailing could provide evidence-based arguments for anticoagulation, favoring benefits over risks for the individual patient. Through interdisciplinary team meetings, consistency in the views of team members is central when they interact with patients and families over any controversial or potentially divisive issues.

Medical care review of medications in conjunction with clinical pharmacists and other team members is an important contribution of leadership in the ACE unit. The team may refer to guidelines for the appropriate prescribing of potentially inappropriate medications or high-risk medications documented in evidence-based literature reviews. Likewise, diagnostic and therapeutic procedures are considered with respect to necessity and consistencies with the patient’s goals and preferences of care as well as established clinical guidelines. In the first ACE units, particular focus was placed on psychoactive and anticholinergic medications. Subsequently, the creation of an ACE tracker has served to efficiently inform the interdisciplinary team of functional and clinical issues such as potentially inappropriate drug prescribing relevant to the acute hospital care of patients [40]. The ACE tracker provides a spreadsheet of information about each patient including length of hospital stay; falls risk; ischemic injury risk; prescribing or administering of potentially inappropriate medications, notably antipsychotic medications; and reports mobility risk score and ADL status, presence of an advanced directive, and history of hospital readmission. The ACE tracker is invaluable as a quality improvement tool and for documenting high-risk characteristics of patients and their treatments.

Medical care review includes the implementation of protocols to minimize the adverse effects of selective procedures ranging from environmental interventions such as a safe room, deployment of family members or sitters at the patient’s bedside, guidelines for use of parenteral alimentation, and prevention and treatment of ischemic injuries [15,17]. Information from the nurse and attending physician are reflected upon and enhanced by further evaluations and recommendations made during team meetings.

## 3. Results: Evidence of ACE Unit Effectiveness

Three randomized clinical trials of ACE units have been published. Each clinical trial maintained fidelity to the original ACE unit model but differed with respect to primary objectives, protocol documentation, outcome measures, and clinical settings. Two of the studies were conducted at a tertiary care teaching hospital in Cleveland Ohio, while the other study was conducted at a community hospital in Akron, Ohio.

### 3.1. University Hospitals of Cleveland

The first randomized trial of the ACE unit enrolled 651 patients 70 years of age or older who were admitted to general medical units for treatment of acute illnesses [2]. The randomization occurred while patients were in the emergency department. Patients were randomly assigned to receive usual care on general medical units or to receive care on the ACE unit. The ACE unit allocated 15 beds to geriatric patients but only a fraction of patients were enrolled in the clinical trial. Patients and their family members when indicated were interviewed by trained research assistants who gathered all clinical data with respect to baseline and admitting functional status, cognitive assessment, mobility assessment, caregiver strain, living situation, mood, and perceived health. Clinical data were obtained from medical records: hospital costs as estimated by the hospital’s cost accounting system, length of hospital stay, and interviews conducted by telephone by trained research assistants following hospital discharge. Deaths were confirmed by review of the national death index and hospital medical records. The primary outcome was performance of five ADL from admission to discharge. Patients admitted to the ACE unit received usual care under an attending and resident team. Nursing budgets were similar on usual care and ACE units. However, substantial changes were made to the physical environment of the ACE unit and included a common area for patient socialization, meals, and limited exercise. Independent performance in basic ADL (bathing, dressing, transferring, toileting, and eating) were ascertained as unique ADL items. 

By the time of discharge, patients in the intervention group were significantly better in their performance of ADL compared to patients in the control units. They were also significantly less likely to be worse in their performance of ADL. A small but not statistically significant difference in length of stay and hospital costs were noted. Patients admitted to the ACE unit were significantly less likely to transition to postacute facilities versus home compared to patients admitted to usual care units. A cost analysis showed that total costs for patients on the ACE unit, accounting for the additional expenses of renovation of the units and salary support of personnel, were not significantly different from usual care. No differences in postacute mortality or ADL function at 90 days were noted between the control and intervention groups.

### 3.2. Akron City Hospital

The second randomized clinical trial included a larger sample and longer-term follow-up. Again, the study compared clinical outcomes between patients admitted to usual care medical-surgical units and patients admitted to the ACE Unit. Many of the patients on the ACE unit were managed by private physicians without resident teams, in contrast to the first ACE study. A similar process of interdisciplinary providers and team-based care was implemented. Eligible patients were identified in the emergency department and 1531 patients were enrolled in the study [41]. The ACE intervention was carried out on a 34–bed unit that was renovated to maintain fidelity to the first study. In addition, a survey was conducted among patients, caregivers, physicians, and nurses to evaluate satisfaction with care on the ACE unit compared to previous hospital experiences. The primary outcome was the performance of five ADL from baseline to discharge. No statistically significant differences were seen among patients receiving care on the ACE unit versus usual care. However, a composite outcome of ADL decline from baseline or nursing home placement occurred less frequently in the intervention group at discharge and during the year following discharge. Satisfaction with care was higher for patients and providers in the intervention group than the usual care group. Mobility score was higher in the ACE treated cohort than usual care cohort. Process of care measures, including absence of physical restraint use, were significantly better on the ACE unit than usual care units. Costs of hospitalization were similar in the intervention and control groups.

### 3.3. Second Clinical Trial at University Hospitals of Cleveland

The third randomized clinical trial enrolled 1632 patients and maintained fidelity to the key elements of the ACE program [42]. The larger sample size enabled better assessment of the effect of ACE on hospital length of stay and cost outcomes. During the clinical trial, the usual-care units were relocated to a new hospital tower that resembled the physical environment of the original ACE unit, perhaps contaminating the intervention. The clinical trial found no effect on ADL functioning between ACE and usual care units. However, the length of hospital stay was significantly shorter for patients admitted to the ACE unit compared to patients admitted to usual care. There was also a significantly lower cost of care per diem and total for ACE versus usual care patients. Impressively, substantially lower inpatient total costs per patient were observed while maintaining independent functioning of patients in their performance of ADL and not increasing hospital readmission rates. Although hospital payments were not measured, the shorter length of stay implies greater revenue versus costs for the hospital, on the ACE Unit.

### 3.4. Summary of ACE Unit Trials

The three clinical trials suggest greater efficiency of patient care at lower cost and with trends supporting the hypothesis that functional disability as measured by the performance of ADL can be reduced by patient admission to an ACE unit. A systematic review and meta-analysis of acute geriatric unit care for elders demonstrated significant benefits of shorter length of hospital stay, fewer discharges to a nursing home, and lower costs [43]. 

A retrospective cohort study of hospitalists’ patients aged 70 years or older spending the entirety of their hospitalization in either the ACE or usual care units examined variable and direct costs of an interdisciplinary ACE unit compared to a multidisciplinary usual care (UC) unit [44]. Total costs were significantly lower for the ACE unit patients, as were 30-day hospital readmissions. The study supports the earlier ACE unit studies showing the lower costs of an ACE unit compared to usual care units. 

Other models of acute hospital care have emerged and appear to be promising or effective alternatives to unit-based care [45]. ACE units share many objectives in common with the Hospital Elder Life Program (HELP) for Prevention of Delirium (https://www.hospitalelderlifeprogram.org), a model of care designed to prevent incident delirium in medically-ill hospitalized older adults and that employs elder life specialists and volunteers to conduct the intervention [46]. The HELP program is cost-effective when targeted at patients at moderate risk of incident delirium. It has been widely disseminated and has been integrated in some hospitals with ACE unit care. 

In clinical trials of ACE units, patients enrolled were aged 70 years and older and admitted to general medical units. Patients were not enrolled from surgical units or intensive care units, although some were transferred to such units when warranted. There is limited data available to address the effectiveness of ACE units on patients younger than 70 years of age or to answer the question of whether all adult patients admitted to general medicine units would benefit from interventions described as key features of the ACE unit. If cost analyses were similar in younger patients, and given the low cost of the intervention, potentially all acutely ill patients admitted from home could benefit from an intervention to prevent functional decline.

Finally, no study has identified a subgroup of patients who are most likely to benefit from admission to an ACE unit. Again, the low cost of the intervention makes targeting less relevant and gives great flexibility to hospital systems and providers to select for admission the types of patients they consider to be most likely to respond to the ACE intervention. The original ACE unit studies attempted unsuccessfully to exclude nursing home patients believing that they were least likely to benefit from admission to ACE units as they were often totally disabled at baseline in their performance of ADL.

4.0. Barriers to ACE Unit Dissemination; “If ACE Units Are So Great, Why Aren’t They Everywhere?” “It’s not sexy stuff. In fact, it’s really quite routine care, involving communication and discharge planning that should be the norm for all hospitals trying to do what’s right for their patients” [47]. 

**Figure 1:**
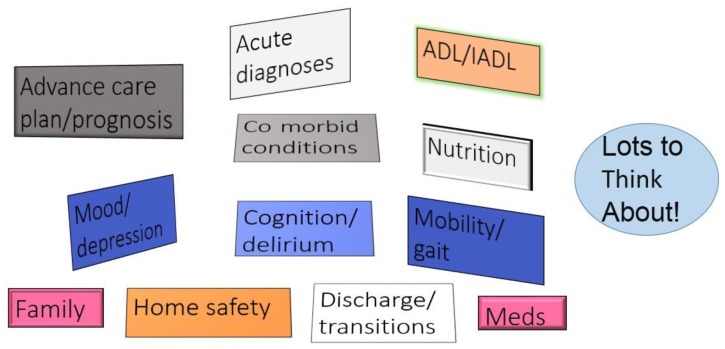
Complexity of hospitalized older adults. Acute care for elders provides structure to the assessment of older adults during hospitalization.

In fact, dissemination of ACE as an intervention has been limited. As a philosophical model of care for hospitalized older adults, the ACE intervention focuses on function, the physical environment, the expertise of an interdisciplinary team, and acute and chronic illnesses affecting postacute needs. In contrast, the biomedical model places limited focus on environmental issues, but focuses on physicians’ direct medical decision-making and the acute illness [48]. The philosophical model of ACE requires transformation of the culture of usual care. Without financial incentives to hospitals under fee for service Medicare reimbursement, chief executive officers and corporate leaders, highly concerned with revenue streams, have little incentive to implement programs that are not immediately revenue generating even though, as the data demonstrate, ACE improves quality and reduces total costs. The benefits of ACE are not readily apparent to hospital leaders despite the relevance of other regulatory and financial incentives to reduce 30-day readmissions and prevent hospital-acquired conditions, or to reduce the incidence of geriatric syndromes including delirium and falls that have shared predisposing factors that ACE and HELP programs can reverse or prevent. The unintended emphasis on a single unit since publication of the first ACE unit intervention conflicts with the reality that general hospitals are increasingly admitting older patients “everywhere,” raising questions about the utility of investing in a single unit. However, the ACE intervention was always considered scalable with the intent that successful components of the intervention would be adopted by other hospital units [1]. There are a few examples of scaled ACE unit programs in the United States, but evidence-based research is limited [48,49]. Perhaps most significantly, there is a misperception that the ACE model of acute-care is a highly complex (hence challenging) intervention, whereas the truth is that older patients are complex (and challenging) and require a different model of care (including an interdisciplinary team to collaboratively evaluate and manage the myriad concerns of complex older patients) than is generally prevalent today (Figure 1). 

This misperception, though, also fails to recognize that ACE is both a continuous quality improvement process and a patient safety program. When fully mature, ACE produces a well-trained, skilled, and competent set of healthcare professionals who are better prepared to provide efficient and less costly medical care to the group of patients who are most complex and likely to benefit from the comprehensive care of the ACE program. As healthcare delivery in the United States continues to transform and managed care programs, accountable care organizations, and bundled payments for care improvement bring value-based care and potential shared savings programs to organizations and hospital systems, ACE could be recalled with favor [50,51]. 

Another concern is the shortage of geriatricians in the United States, especially with a career focus on acute care. A small but growing number of hospitalists are trained in geriatrics and are committed to improving quality of care such as that seen in ACE programs. The geriatrician’s role is primarily medical care review and as an educator of the interdisciplinary team and staff. Many ACE units can be directed by advanced care nurses. Lack of training of health care professionals in gerontology and geriatric practice is problematic. Healthcare providers who are not trained in geriatrics may not realize what they are overlooking or make diagnostic errors. Finally, there is no single measure of activities of daily living that is measured by all hospitals. Reimbursement to hospitals and physicians is not based on the functional status of patients, nor of change in their mobility during hospitalization. Until there is consensus about the appropriate tool to measure ADL, and regulatory changes reward hospitals to account for changes (improvements) in physical functioning and mobility, dissemination of ACE units will be highly limited. The evidence is clear: functional disability is predictable and at least partially preventable, and incurs significant deficits persisting well after hospitalization in previously functionally independent people [52,53]. This phenomenon of persistent morbidity and functional disability is labeled as a “post-hospital syndrome” accompanied by impaired quality of life of older people [54].

## 4. Conclusions and Final Comment

The ACE unit model underwent changes during the clinical trials that mirror current trends in acute hospital care of adults. Physical environments are becoming more “ACE-like”, clinical pharmacists are monitoring the prescribing of potentially inappropriate medications, financial incentives are in place to reduce hospital acquired conditions that are relatively more common in older adults (falls with injury, ischemic injuries, and catheter associated urinary tract infections), and planning for transitions of care is more commonly performed. Time and sheer numbers of older adults are on the side of our seniors: acute hospital care will improve and ACE is the vision.

## Figures and Tables

**Table 1:** Prepared and Safe Hospital Environment.

**Goal**: Standardize safe furniture and equipment placement in the patient room and public thoroughfares to prevent falls and injuries and to optimize patient self-care.
**GENERAL PRINCIPLES FOR PATIENT ROOM**:
Note: Americans with Disabilities Act (ADA) requires 10% of Acute Care beds comply with ADA standards
❖ CONTENTS per Patient Bed ○ One patient chair (with armrests) ○ One visitor chair (armrests preferred). If additional visitor chairs, consider using folding chairs in order to remove or fold away when not in use.
Note: If only one chair can fit into the room, the priority is the patient chair.
Also, recommendation is that 20% of acute care beds to be equipped with bariatric furniture.
○ One night stand ○ One over-bed table ○ Telephone (type that mounts onto side rail preferred) ○ Patient waste can ○ Two staff waste cans (regular trash and hazardous waste) ○ No linen carts (holder on wall with linen bags preferred) ○ These items are needed only if patient is using them ▪ IV pole ▪ Bedside commode with toilet paper holder mounted on side ○ Electrical outlets every 12 feet (standard) can be adapted to equipment and usage needs in the patient room ○ Furniture and sinks with rounded edges (minimizes injury if patient falls)
❖ SPACING/PATHWAYS ○ Clearance space of 3 feet exists around the bed, except at the headwall (ADA). Primarily applies to stationary furniture/equipment. Movable furniture is permitted within this space. ○ Minimum 3 feet between patient beds in semi-private rooms (ADA) ○ Vertically, anything protruding from the wall, within a zone of 80 inches from the floor, must be < to 4 inches, except at the headwall of the bed (ADA) ○ Clear pathway from patient bed to bathroom and entrance/exit to room
❖ SAFE BED EXIT ○ Safe bed exit side is identified and located on patient’s side of preference, or dominance, especially if a functionally limiting clinical condition exists (such as weakness due to stroke). If no patient preference, the default for safe exit is the side of the bed closest to the bathroom. ○ Safe exit side of bed is visually noted in the patient’s room ○ Items on safe exit side include: ▪ Night stand (within reach)
❖ IV Pole (if being used by patient) ○ Bedside commode (if being used by patient) ○ Items NOT on safe exit side include: ▪ Over bed table ▪ Chairs (patient and visitor) ▪ Patient’s garbage can
❖ GRAB BARS ○ Continuous grab bars or handrails available along walls, except where there is affixed, stationary furniture.
Note: This decreases room space by 3 inches on every side there is a grab bar. May want to consider furniture placement as an alternative.
❖ FURNITURE/EQUIPMENT ○ Patient chair is designated as such and has armrests ○ Rounded corners on furniture or bumper guards on edges ○ Assistive equipment and call bell is within patient’s reach ○ Lever handles on doors, no doorknobs (ADA) ○ Divider curtains between beds pull all the way back to the wall ○ Electrical cords bundled and kept away from walking paths
❖ LIGHTING ○ Diffuse lighting that projects vertically ▪ Perforated screen covers to minimize glare if patient passes underneath on a carrier Under bed light that illuminates floor around the bed ○ Low lighting along base of walls in patient room, especially to light path to bathroom and entrance/exit of patient room ○ Light controls on bed rail and on call light controller
❖ BATHROOM ○ No tub ○ Walk-in/wheel-in shower (ADA) ○ Doorway wide enough for patient and equipment (Standard dimensions: patient room an entry door width of 48 inches, bathroom entry width of 36 inches) ○ Continuous grab bars, especially behind and on wall side of toilet (ADA) ○ Flip down bars not recommended for toilet area, instead use wall mounted or toilet mounted grab bar that utilizes a mounting bracket ○ Sinks with no support between sink and floor must meet mounting standards to tolerate patient weight leaning on sink ○ “No Slip“ surface on floor (0.08 slip co-efficient on potentially wet surfaces) ○ Devices available to elevate toilet seat 17–19 inches from floor (ADA) ○ Emergency cord accessible from both toilet and shower (ADA) ○ Curbless shower threshold (ADA) with two drains (one inside shower and one outside shower area) ○ Sensor light in bathroom that automatically turns on when someone enters ○ Glow in the dark toilet seats, or seats with a glowing border to help patient locate it (not necessary if lighting turns on automatically on entry). Nightlight that illuminates toilet area is an alternative.
❖ HALLWAYS 8 foot wide corridors ○ No equipment permanently stored in hallways ○ When in use, equipment placed on one designated side of hall ○ Low glare floors with visual breaks (synthetic surfaces) ○ Handrails on both sides of the hall that are either a different color than the walls, or have built in lighting to provide contrast against the wall ○ Diffuse lighting that projects vertically ○ Mirrors for blind corners ○ “High risk” patient room with adjustable visibility to front of room for monitoring

**Table 2:** Interdisciplinary Team Members, Tasks and Roles.

Member	Tasks/Roles
Physician and/or bedside nurse	Admitting diagnosis or problem: key findings Relevant past medical history Treatment plans Anticipated length-of-stay and postacute site of care
Bedside nurse (report)	Assess baseline and current functional status: ADL, mobility, mood/affect, cognition, living situation, social support, nutritional status (role shared with physician) Implement preventative/restorative protocols
Care coordinator/social worker	Identify resources (caregiving, finances, options) Coordinate discharge (transitions) options Order durable medical equipment
Clinical pharmacist	Assess medication appropriateness (potentially inappropriate medications) (shared role with physician) Plan for monitoring of high risk medications
Physical therapist	Mobility assessment (shared role with bedside nurse) Transfer and gait assessment with recommendations Determine need for skilled services (rehabilitation)
Occupational therapist	Assess need for ADL devices/aids Evaluate physical functioning Determine need for skilled services (rehabilitation)
Dietitian	Assess baseline nutritional status Offer dietary recommendations Work with speech therapy in assessment of oral feeding
Summary: Interdisciplinary team	Estimate functional trajectory Estimate length of hospital stay Estimate postacute requirements Review quality of care and safety Plan for care transitions
Patient and family (medical power of attorney)	Review goals of care, personal preferences, advance directives Engage in self-care Share decision-making with ACE team
